# Comparison of Intravitreal Aflibercept and Ranibizumab for Treatment of Myopic Choroidal Neovascularization: One-Year Results—A Retrospective, Comparative Study

**DOI:** 10.1155/2019/8639243

**Published:** 2019-10-31

**Authors:** Burak Erden, Selim Bölükbaşı, Emine Baş, Akın Çakır

**Affiliations:** University of Health Sciences, Okmeydanı Education and Research Hospital, Retina Department, Istanbul, Turkey

## Abstract

**Purpose:**

To compare one-year treatment outcomes of intravitreal aflibercept (IVA) and intravitreal ranibizumab (IVR) for treatment of myopic choroidal neovascularization (mCNV).

**Methods:**

The medical records of a total of 30 eyes diagnosed with mCNV and underwent IVA or IVR treatment for a minimum one-year follow-up were studied retrospectively. All the subjects had an axial length >26 mm and received a 1 + PRN (pro re nata) regimen IVA or IVR. Best-corrected visual acuity (BCVA) and central macular thicknesses (CMT) on optical coherence tomography were evaluated before and after treatment.

**Results:**

There were 12 eyes in IVA group, with a mean age of 60.0 ± 10.2 years. The mean BCVA significantly improved from baseline 1.54 ± 0.76 to 0.85 ± 0.61 and the mean CMT significantly decreased from baseline 384.3 ± 119.1 *μ*m to 305.9 ± 75.4 *μ*m to 305.9 ± 75.4 *p* : 0.024 and *p* : 0.024 and *μ*m to 305.9 ± 75.4 *μ*m to 305.9 ± 75.4 *p* : 0.024 and *p* : 0.024 and *p* : 0.024 and *p* : 0.024 and

**Conclusions:**

Both IVA and IVR treatment modalities resulted in similar anatomical outcomes but IVA had better visual outcomes in treatment of mCNV.

## 1. Introduction

Myopic choroidal neovascularization (mCNV) is the major vision-threatening complication of pathologic myopia [1, 2]. The pathologic myopia is characteristic with a myopic refractive error >6.0 diopters and axial length >26 mm and usually associated with typical degenerative changes of the fundus. The incidence of mCNVs ranges from 5.2% to 11.3% among the population with pathologic myopia [3, 4]. If left untreated, the long-term prognosis of this clinical entity is devastating. In 5 years, 88.9% of the pathologic myopic patients with mCNV can experience severe visual decline with a best-corrected visual acuity (BCVA) of 20/200 or less and in 10 years this proportion might increase up to 96.3% [5]. The CNV-related macular atrophy and fibrosis following the CNV regression is usually the major cause of the permanent visual loss [6].

Nowadays, the standard of care for mCNV is the intravitreal administration of anti-vascular endothelial growth factor (anti-VEGF) agents such as ranibizumab and bevacizumab, which have better clinical results compared to previous treatment modalities such as PDT leading these agents to the first-line therapy for mCNVs [7, 8]. Several clinical comparisons and meta-analysis were conducted to find out which anti-VEGF agent had preferable clinical efficacy [9–11]. To the best of our knowledge, the current study is the first head-to-head comparison of aflibercept and ranibizumab in the treatment of mCNV patients.

## 2. Material and Methods

This retrospective and single-center comparative study was carried out in the Retina Department of University of Health Sciences, Okmeydanı Education and Research Hospital. It was approved by the Local Ethical Committee of Okmeydanı ERH and the procedures adhered to the principles of the Declaration of Helsinki and Good Clinical Practice guidelines. Informed consent was signed by all the participants prior to each intervention.

The medical records of 43 eyes of 41 patients diagnosed with myopic CNV and treated at our retina department between January 2016 and February 2019 were reviewed retrospectively. Patients treated with either 2 mg/0.05 ml intravitreal aflibercept (IVA) or 0.5 mg/0.05 ml intravitreal ranibizumab (IVR) and who had a minimum follow-up of one year were included. None of the patients had any kind of treatment including the intravitreal anti-VEGFs within the previous 6 months before the administration of anti-VEGF drug at our clinic. The presence of CNV secondary to different etiologies other than pathologic myopia, presence of uncontrolled glaucoma, history of a prior macular photocoagulation or photodynamic therapy, iris neovascularization or vitreous hemorrhage, and the history of thromboembolic events were the exclusion criteria. Pseudophakic patients with a history of uneventful cataract surgery were not excluded from the study population. All the eligible patients had an age >18 years, axial length more than 26 mm, and high myopia with a spherical equivalent greater than 6.0 diopters (in phakic eyes). Finally, 30 eyes of 30 patients were enrolled into the current study.

The myopic CNV was diagnosed based on the findings of a detailed ophthalmologic examination, spectral domain optical coherence tomography (SD-OCT) imaging, and fundus fluorescein angiography (FFA; Visucam500, Carl Zeiss Meditec, Jena, Germany), which were performed to all the subjects prior to treatment initiation. An A/B-mod ultrasound device (HiScanTouch, Optikon, Italy) was utilized by two different retinal specialists (BE, SB), and mean values were taken into account to determine the axial length of each individual patient. SD- OCT scans (Spectralis, Heidelberg, Germany) and detailed ophthalmologic examination including best-corrected visual acuity (Snellen), anterior biomicroscopy, Goldmann applanation tonometry, and dilated fundoscopy were all implemented at all visits.

IVA or IVR administrations were performed under sterile conditions and topical anesthesia on a 1 + pro re nata (PRN) regimen followed by monthly or bimonthly prescheduled visits for at least 12 months. Patients in both arms were followed up and treated strictly on a monthly basis in the initial treatment interval, but after reaching the inactive phase of the mCNV, the follow-up regimen turned into a bimonthly basis. Our objective retreatment criteria in the study period were as follows: (1) a decline in BCVA greater than one Snellen line compared to the previous visual examination; (2) central macular thickness (CMT) increase ≥50 *μ*m from the previous OCT examination; (3) persistent or recurrent intraretinal or subretinal fluid on SD-OCT; and (4) persistent or recurrent submacular hemorrhage on the fundus examination. Besides, a subjective complaint about visual function—such as increase of metamorphopsia—combined with the presence of any intra- or subretinal fluid or new accumulation of highly reflective dots on SD-OCT foveal scans was an additional retreatment criterion in the study period.

The BCVA and CMT values were compared intra- and intergroup at baseline and months 1, 2, 3, 6, and 12. Statistical analyses were carried out using IBM SPSS 22.0 software (SPSS Inc., Chicago, IL). All values were expressed as means and standard deviation. The data were evaluated for normality using both visual (histograms, probability plots) and analytical methods (Kolmogorov-Smirnov, Shapiro-Wilk test). Since the distribution of CMT values were found to be normal, inter- and intragroup CMT changes between baseline and following time points were assessed with repeated measures test of Anova. BCVA values, however, especially in the IVR group, were not distributed normally; hence Mann–Whitney *U* test was used for intergroup comparisons at baseline and at time points in follow-up, and Wilcoxon signed rank test was utilized for intragroup analyses. A *p* value of less than 0.05 was considered to be statistically significant.

## 3. Results

A total of 30 eyes were finally enrolled into this study. While 12 eyes constituted the IVA group, 18 eyes were included into the IVR group. There were no significant differences in means of gender distribution and mean age between both groups (*p*=0.574 and *p*=0.677, respectively). The mean ages of IVA and IVR groups were 60.0 ± 10.2 and 57.4 ± 13.1 years, respectively. At FA examination, all mCNVs revealed early hyperfluorescence with leakage in later phases of the angiogram. None of the patients had intra- or subretinal hemorrhage at baseline. All CNVs had a subfoveal localization confirmed with both FA and baseline OCT scans. The mCNVs' compositions in both groups were similar (*p*=0.79). In IVA group, 7 cases out of 12 (58%) had mixed type (type I + II) CNVs; in IVR group, 11 eyes of 18 (61%) were complicated with mixed type CNVs, and in the rest of both groups, the mCNVs were diagnosed as type I (occult) membrane. Both groups were found statistically comparable in clinical parameters such as mean axial length and spherical equivalents of phakic eyes (28.4 ± 2.3 versus 29.3 ± 3; 9.4 ± 1.9 versus 9.6 ± 1.3). 3 eyes of 12 (25%) in IVA group and 4 eyes out of 18 (22%) in the IVR group had a history of a single intravitreal treatment (ranibizumab or bevacizumab) beyond the six months before our study enrollment.  Table 1 demonstrates the demographic and clinical baseline characteristics of both study groups.

**Figure 1:**
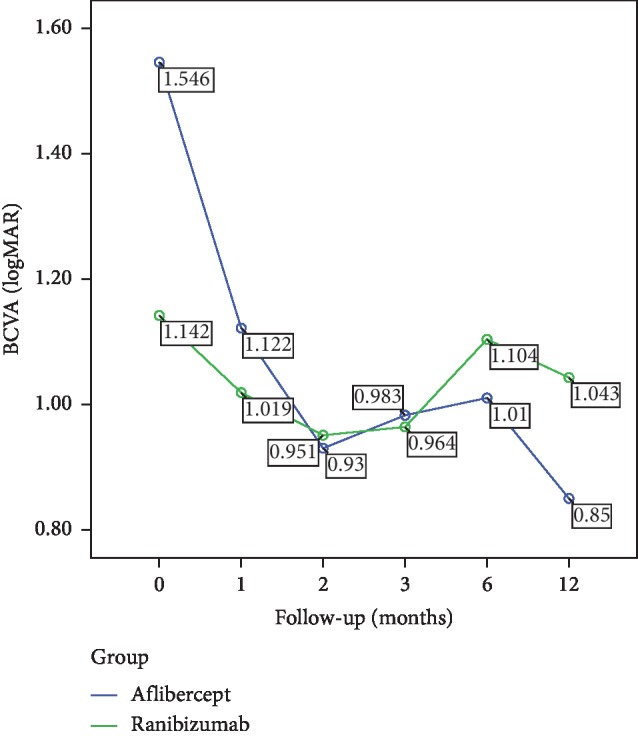
The mean BCVA increased in the IVA group significantly even at Month 2 (*p*=0.04) and the visual improvement continued till the final visit (*p*=0.024). The IVR group, however, maintained basically its baseline BCVA levels throughout the study period (*p*=0.345).

The mean BCVA in the IVA group significantly improved from baseline 1.54 ± 0.76 to 0.85 ± 0.61 at Month 12 (Wilcoxon signed rank test; *p*=0.024). In the IVR group, however, the mean BCVA changed from baseline 1.14 ± 0.90 to 1.04 ± 0.93 at Month 12 insignificantly (Wilcoxon signed rank test; *p*=0.345). The intergroup analysis revealed a significant superiority of IVA group over IVR group in means of visual gain at the final 12th month visit (0.69 versus 0.09; Mann–Whitney *U* test, *p*=0.006) (Figure 1).

**Figure 2:**
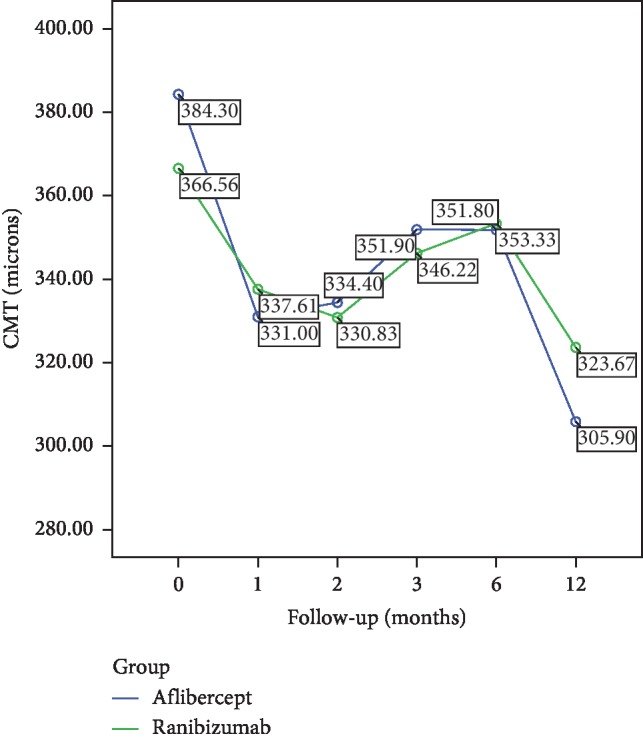
The CMT reduction of both groups had similar outcomes at all time points with no significant intergroup differences (*p* : 0.704).

**Figure 3:**
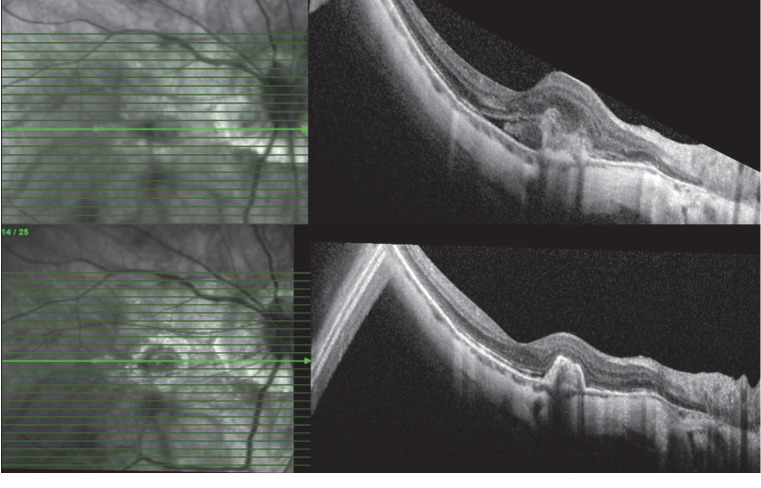
In the case of the IVA group, the mCNV‐associated subretinal fluid totally regresses following two monthly IVA injections; the type I component of mCNV is still present at the following visit.

**Figure 4:**
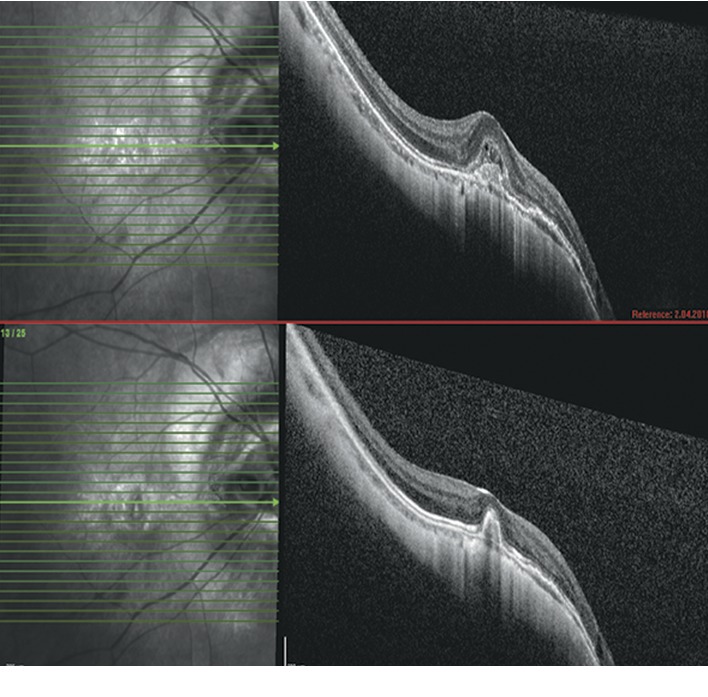
In one demonstrative case of the IVR group, the regression of the subretinal fluid after two consecutive monthly IVR administrations is clearly seen in a comparison of foveal OCT scans.

The mean CMT value significantly decreased from baseline 384.3 ± 119.1 *μ*m to 305.9 ± 75.4 *μ*m at Month 12 in the IVA group (*p* < 0.001). The mean CMT significantly decreased from baseline 366.5 ± 102.3 *μ*m to 323.6 ± 103.6 *μ*m at Month 12 after IVR therapy (*p* < 0.001). There was no significant difference between the groups regarding changes in CMT during the study period (*p* : 0.704) (repeated measures of ANOVA) (Figure 2). The mean number of intravitreal administrations in IVA and IVR groups was 2.9 ± 1.6 and 2.5 ± 1.6 (*p*=0.605), respectively. The mean number of visits in the study period revealed also no significant difference between treatment groups (6.3 versus 6.8, *p*=0.73). At the baseline, OCT examination in 10/12 (83%) cases of the IVA group had intraretinal and in 6/12 (50%) cases subretinal fluid. In the IVR group, 14/18 (77%) eyes had intraretinal and 5/18 (27%) subretinal fluid associated with the active mCNV. At the final visit, only 2 eyes (16%) in the IVA group had residual intraretinal fluid. Subretinal fluid was regressed in all cases of the IVA cohort (Figure 3). At the final visit of the IVR group, the presence of intraretinal fluid was seen in only 3 (16%) eyes, and subretinal fluid was totally regressed in all cases (Figure 4). No systemic or ocular adverse event was reported within the follow-up associated with intravitreal treatments.

## 4. Discussion

The mCNV secondary to pathological myopia had been a challenging clinical entity for many years in field of retinal diseases. The pioneering clinical studies with off-label agent bevacizumab [12, 13]—first intravenous and then intravitreal—were followed by studies with ranibizumab [14] and aflibercept [15] and consequently led these anti-VEGF therapies into the first-line treatment modality of mCNV patients. Still, there is a lack of randomized head-to-head comparisons between different anti-VEGF agents in this area. The question which on-label anti-VEGF agent should be preferred by the clinicians remains controversial, since a direct comparison between ranibizumab and aflibercept has not been reported yet.

The first report of the randomized, double-masked, multicenter RADIANCE trial (*n*: 277), e.g., demonstrated that its 0.5 mg ranibizumab only treatment arms led to superior visual gain results over PDT (10.5 and 10.6 versus 2.2 ETDRS letters) at Month 12 [16]. The pivotal MYRROR trial, on the other hand, emphasized the efficacy of 2 mg intravitreal aflibercept (*n*: 90) in a comparison against the sham group (*n*: 31) and pointed to the importance of early intervention since the sham group did not catch the final visual gain of IVA groups (13.5 versus 3.9 ETDRS letters) despite the late rescue therapy following the primary end-point week 24 [15]. In both clinical trials, the visual improvement—with either aflibercept or ranibizumab—reached apparently its plateau levels at Month 3 which was consistent to our visual outcome findings. Likewise, in IVA and IVR groups of our study population, the increase of BCVA values continued up to Month 3 and thereafter a visual stabilization was observed in further follow-up. In RADIANCE trial, the investigators reported in their 1 + PRN ranibizumab arm a median number of IVR injections as 2, comparable with our mean injection numbers in both IVA and IVR groups, 2.9 and 2.5, respectively.

Our anatomical results in means of CMT reduction proceeded the highest visual improvement time point (Month 3) and started earlier such as at the 1st and 2nd month visits in both study groups. This phenomenon was also consistent with previous reports. In MYRROR trial, e.g., the CMT was reduced rapidly following the as-needed aflibercept treatment reaching the plateau levels between week 4 and week 8. Such an early improvement of CMT values was also reported in a recent retrospective clinical study comparing aflibercept to ranibizumab in mCNV patients [10].

Several studies compared previously in small sample case series anti-VEGF agents against each other in terms of efficacy—especially ranibizumab versus bevacizumab [17–19]. Gharbiya et al., e.g., compared 1.25 mg intravitreal bevacizumab (IVB) with 0.5 mg ranibizumab on a priori as-needed basis in a prospective randomized clinical trial (RCT) with a follow-up of 6 months [17]. They reached comparable visual and anatomical outcomes with similar overall number of injections (2.81 IVR versus 2.44 IVB) in 6-month study period. Later, Iacono et al. compared these two agents in an RCT with a longer follow-up (18 months) [18]. At the end of their study period, they reported similar functional outcomes in both arms but the IVR arm achieved this improvement with a significantly smaller number of injections (2.56 IVR versus 4.72 IVB). In a retrospective study of 24-month follow-up, Lai et al. found superior visual results in their IVR subgroup with the same number of treatments (3.8 IVR versus 3.8 IVB) [19]. All these above-mentioned reports supported the relative superiority of ranibizumab over bevacizumab to some extent. According to the findings of these comparative studies, IVR resulted in better visual outcomes with the same number of injections or IVR treatment resulted in comparable visual improvement with significantly lower number of injections. However, more additive data deriving from larger series are needed to support these conclusions.

In a more recent comparative retrospective study, Wang et al. reported that both IVB and IVA treatments were equally effective in means of CMT reduction and visual gain at the end of the study period (Month 12) [10]. The IVA arm underwent significantly less injections (2.11 ± 0.41 versus 3.23 ± 0.38; *p*=0.01) than IVB arm on an as-needed regimen. Their study design was pretty similar to ours including the retreatment criteria. Our comparison between IVR and IVA arms revealed no statistical difference in means of CMT reduction and number of injections needed, but a significant better visual improvement was detected in favor of our IVA arm at the final visit. The main reason of this superior potency of aflibercept might be its broader antagonistic mechanism as it binds as a VEGF-trap all isoforms of VEGF and additionally also placental growth factor [20]. Besides, the VEGF suppression times (VST) were compared by Fauser and Muether in neovascular age-related macular degeneration (nAMD) treatment and they reported that aflibercept had significantly longer VST compared to ranibizumab (67 ± 14 d versus 34 ± 5 days; *p* < 0.001) [21]. Indeed, this longer suppression combined with higher pharmacological efficacy may lead to superior anatomical outcomes with less frequent treatment intervals, e.g., in treating patients with pigment epithelial detachment secondary to nAMD and refractory to ranibizumab [22]. In a recent meta-analysis, Seguin-Greenstein et al. concluded that treatment resistant nAMD patients benefit from switching to aflibercept in anatomical and even functional parameters [23]. In the present study, we also found that IVA has a significant superiority in visual recovery of mCNV patients, but regarding the heterogeneity of this disease and our small sample size of each study arm, our results should be evaluated with caution.

Our study has clearly several limitations. First, with its retrospective design, the absence of any randomization reduces the power of our clinical results to some extent. Secondly, clinical outcomes should be interpreted carefully due its small sample size. However, it has also its strengths; any statistically significant differences were not found in demographic and clinical baseline data between the two groups and it is reflecting clinical results in real life settings. Additionally, this pilot study is the first clinical comparison between aflibercept and ranibizumab in mCNV treatment. According to our results, aflibercept proved itself more effective especially in visual gain aspect, but future studies, preferably randomized clinical trials with larger sample sizes, have to be conducted for further comparative evaluation of these two on-label anti-VEGF drugs in this clinical entity.

## Figures and Tables

**Table 1:** The baseline clinical and demographic characteristics of both groups were found statistically comparable in all parameters.

	*Aflibercept no.: 12*	*Ranibizumab no.: 18*	*pvalue*
Age (years), mean ± SD	60.0 ± 10.2	57.4 ± 13.1	0.574
Gender, *n* (%), female	8 (66.6%)	13 (72.2%)	0.677
Lens status, *n* (%), phakic	5 (41.6%)	9 (50%)	0.434
Baseline BCVA, mean ± SD (logMAR)	1.54 ± 0.76	1.14 ± 0.90	0.172
Baseline CMT (in *μ*m), mean ± SD	384.3 ± 119.1	366.5 ± 102.3	0.697
AL (in mm), mean ± SD	28.4 ± 2.3	29.3 ± 3.1	0.321
Treatment naïve status (%)	75%	78%	0.81
Mean number of treatments in one-year follow-up ± SD	2.9 ± 1.6	2.5 ± 1.6	0.6
The types of mCNVs	58% mixed (type I + II)	61% mixed (type I + II)	0.79
SE^*∗∗∗∗∗*^ mean ± SD in diopters (phakic eyes)	−9.4 ± 1.9	−9.6 ± 1.3	0.512

BCVA, best-corrected visual acuity; CMT, central macular thickness; AL, axial length; mCNV, myopic choroidal neovascular membrane; SE, spherical equivalent.

## Data Availability

The data used to support the findings of this study are available from the corresponding author upon request.
